# CXCR4-Targeted and MMP-Responsive Iron Oxide Nanoparticles for Enhanced Magnetic Resonance Imaging**

**DOI:** 10.1002/anie.201405442

**Published:** 2014-07-15

**Authors:** Juan Gallo, Nazila Kamaly, Ioannis Lavdas, Elizabeth Stevens, Quang-De Nguyen, Marzena Wylezinska-Arridge, Eric O Aboagye, Nicholas J Long

**Affiliations:** J. Gallo, N. Kamaly, I. Lavdas, E. Stevens, Q.-D. Nguyen, E. O. Aboagye, N. J. Long, Comprehensive Cancer Imaging Centre, Department of Surgery and Cancer, Hammersmith Campus, Imperial College LondonDu Cane Road, London, W12 0NN (UK); J. Gallo, N. Kamaly, N. J. Long, Department of Chemistry, Imperial College LondonSouth Kensington, London, SW7 2AZ (UK); M. Wylezinska-Arridge, Biological Imaging Centre, Medical Research Council (MRC) Clinical Science Centre, Imperial College LondonDu Cane Road, London, W12 0NN (UK)

**Keywords:** click chemistry, imaging agents, nanoparticles, self-assembly, tumor targeting

## Abstract

MRI offers high spatial resolution with excellent tissue penetration but it has limited sensitivity and the commonly administered contrast agents lack specificity. In this study, two sets of iron oxide nanoparticles (IONPs) were synthesized that were designed to selectively undergo copper-free click conjugation upon sensing of matrix metalloproteinase (MMP) enzymes, thereby leading to a self-assembled superparamagnetic nanocluster network with *T*_2_ signal enhancement properties. For this purpose, IONPs with bioorthogonal azide and alkyne surfaces masked by polyethylene glycol (PEG) layers tethered to CXCR4-targeted peptide ligands were synthesized and characterized. The IONPs were tested in vitro and *T_2_* signal enhancements of around 160 % were measured when the IONPs were incubated with cells expressing MMP2/9 and CXCR4. Simultaneous systemic administration of the bioorthogonal IONPs in tumor-bearing mice demonstrated the signal-enhancing ability of these ‘smart’ self-assembling nanomaterials.

The early detection of primary tumors and metastases is a major clinical challenge, and an emerging approach for targeting imaging agents to tumors is to exploit the changes that occur within the local tumor microenvironment. The matrix metalloproteinase (MMP) enzymes MMP2 and MMP9 have been shown to play an important role in tumor development and metastasis.^1^, ^2^ These MMPs are thus excellent biomarkers for the development of tumor-targeted contrast agents.^3^

In biomedical imaging, MRI is a noninvasive imaging technique that has high spatial resolution and does not require ionizing radiation.^4^ However, MRI suffers from limited sensitivity^5^ and the use of contrast agents is necessary to increase sensitivity and image contrast in MR scans.5a, 6 Superparamagnetic IONPs are widely used in MRI owing to their biocompatible nature and strong effects on *T*_2_ and *T*_2_* relaxation.^7^ To increase the sensitivity of *T*_2_-weighted MRI, several strategies with NPs have been adopted,^8^ however, fewer examples exist that utilize changes in NP size to achieve signal amplification in MR scans.^9^ Larger iron oxide nanoparticles (IONPs) and magnetic nanoparticle aggregates have pronounced magnetic properties,^7^, 9c but are cleared faster from the blood pool by the mononuclear phagocyte system.^10^

We have designed two sets of novel IONPs that only form aggregates within the tumor environment, where self-assembly into larger particles is triggered by cancer-specific MMP biomarkers. The sensitivity of MRI can thus be enhanced through both specific tumor targeting and tumor-associated proteolytic enzyme activity. It is known that magnetic susceptibility increases when NP aggregates are formed and this process also increases the *r*_2_ relaxivity.^11^ The issue of low sensitivity is being tackled,9a,9c, 12 however, the work has either not progressed to in vitro or in vivo stages, or the aggregation process has relied upon electrostatic and non-covalent interactions.

In this work, we utilized copper-free “click” chemistry to achieve NP self-assembly and therefore MR *T*_2_ signal amplification both in vitro and in vivo. Rather than relying on nonspecific processes, we chose to use copper-free click chemistry^13^ to form covalent bonds between the particles. The strategy of targeting the CXCR4 receptor^14^ is crucial and in preclinical studies, this has shown far superior performance compared to passive approaches. CXCR4 levels can be predictive of metastatic potential,14f, 15 and we demonstrate that the EPR effect alone is not enough to highlight tumors.

The general concept is presented in Figure 1; these particles have a surface decorated with peptide ligands that target them to tumor sites. Their structure also contains peptide sequences cleavable by the MMP2/9 enzymes overexpressed in tumors.^3^ The cleavage of the protease-specific peptides exposes either azide or alkyne moieties on the NP surfaces, thereby allowing the particles to undergo a [3+2] cycloaddition reaction. This copper-free chemical reaction leads to self-assembly of the IONPs and the change in particle distribution has an effect on the relaxivity (*r*_2_) of the contrast agent. The relaxivity is higher after assembly,^11^ thus resulting in improved contrast in *T*_2_-weighted MR images. Furthermore, the IONPs are PEGylated, thus leading to improved in vivo bioavailability.^[16]^ The targeting ligand incorporated on the surface of the IONPs is a cyclopentapeptide with affinity for the CXCR4 receptor.^17^

**Figure 1:**
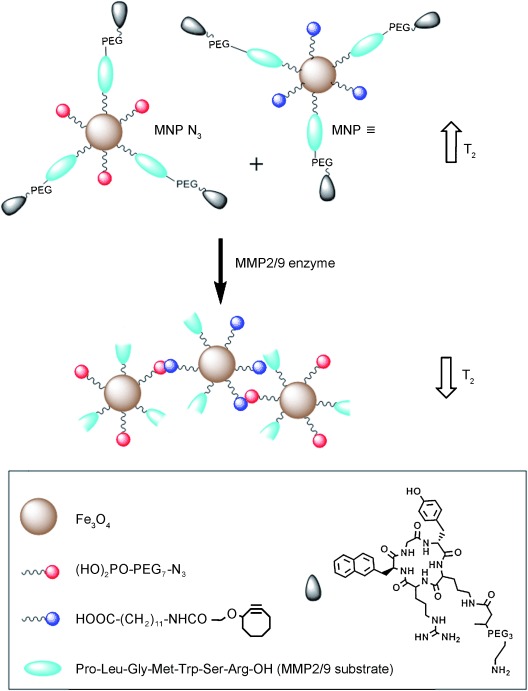
In vitro and in vivo “clicking” NPs. Two complementary IONPs were designed to undergo a bioorthogonal reaction after cleavage by MMP enzymes, which exposes the azide or alkyne moieties on either set of NPs. MNP=magnetic nanoparticle, PEG=polyethylene glycol.

Magnetite (Fe_3_O_4_) was chosen as the core material for the development of the IONPs^18^ and a monodispersed population of oleic acid capped IONPs was prepared according to a reported method.^19^ The particles were fully characterized by using standard techniques (Figures S1, S2 and Data S3 in the Supporting Information). In the next part of the synthetic strategy, a series of sequential surface functionalizations were performed (Figure 2 and Data S4), and the reaction sequences could be monitored by FTIR spectroscopy (Figure S5 and S6) and ^1^H NMR spectroscopy (Figure S7).

**Figure 2:**
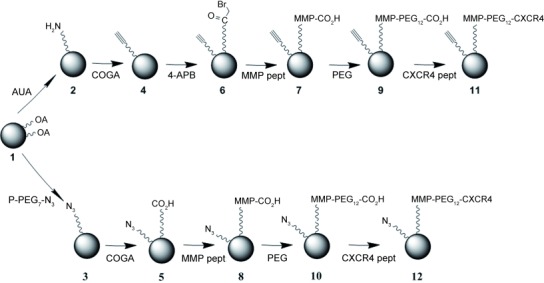
Sequential surface functionalization of synthesized IONPs. AUA=11-aminoundecanoic acid; P-PEG_7_-N_3_=O-(2-azidoethyl)heptaethylene glycol phosphonooxy-ethyl ester; COGA=cyclooct-1-yn-3-glycolic acid; 4-APB=4-azidophenacyl bromide; MMP pept=DNP-Pro-Leu-Gly-Met-Trp-Ser-Arg; P-PEG_7_-N_3_=O-(2-azidoethyl)heptaethylene glycol phosphate; CXCR4 pept=cNal-Gly-d-Tyr-Orn[PEG-NH_2_]-Arg.

In the final step, a targeting cyclopeptide directed against CXCR4 was introduced for specific binding to CXCR4 (to form **11** and **12**), thereby yielding targeted NPs with an average of 10 targeting peptides per particle. The successful preparation of the final ligands was assessed by MALDI mass spectrometry (Figure S8). The simple and repeated chemistry involved in the final stages of the NP preparation (Figure 2) enabled the preparation of different controls, for example, **10** and **13** (Figure 3). The final targeted NPs were very similar in terms of size (Figure 4 A) and surface charge (Table S2 in the Supporting Information), thus suggesting the likelihood of similar circulation times and biodistribution patterns in vivo.

**Figure 3:**
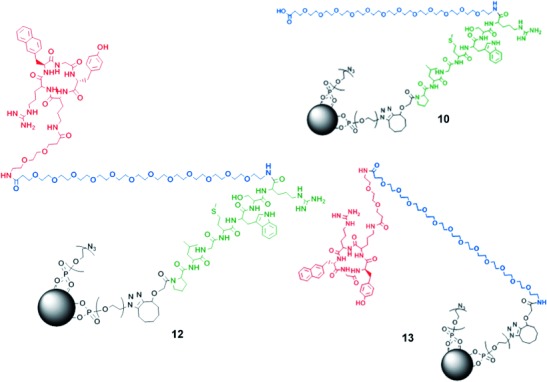
Molecular structures of the final NP (12) and controls; 10=nontargeted NP (“azide family”), 13=NP without self-assembling properties (“azide family”).

**Figure 4:**
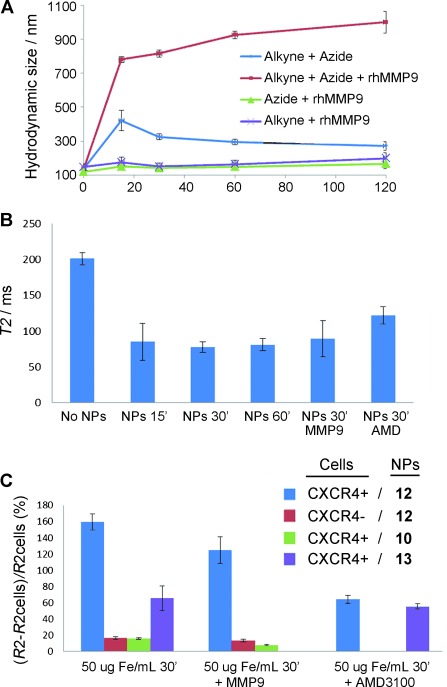
A) Hydrodynamic size measurements. Blue=1:1 mixture of alkyne and azide NPs. Red=1:1 mixture of the alkyne and azide NPs in the presence of hrMMP9. Purple=alkyne NPs in the presence of hrMMP9. Green=azide NPs in the presence of hrMMP9. B) *T*_2_ values obtained from U87.CD4.CXCR4 cells embedded in 1 % agarose gels incubated with a 1:1 mixture of the NPs (50 μg Fe/mL) for different time periods (15, 30, and 60 min), either alone or in the presence exogenous MMP9 or the CXCR4 inhibitor AMD3100 (30 min). C) Δ*R*_2_/*R*_2_ cell values obtained from either U87.CD4.CXCR4 (CXCR4+) or U87.CD4 (CXCR4-) cells incubated with a 1:1 mixture of the NPs (50 μg Fe/mL; either final probe or controls, see Figure 3) for 30 min. The results are given as the mean of three independent experiments ± the standard deviation.

The proof-of-principle for the design was obtained by using hydrodynamic size measurements (Figure 4 A). The two families of NPs were mixed in equimolar concentrations and incubated at 25 °C for 2 h. Hydrodynamic size measurements were acquired at 0, 15, 30 and 60 mins. The data show that after an initial size increase, the sizes tended to return to the original values (proof that no covalent bonds were formed). However, when the incubation was performed in the presence of MMP9, the size of the aggregates increased dramatically over time (6-fold after 15 mins, from 140 nm to 780 nm). The formation of NP aggregates was further confirmed by TEM (Figure S9). Relaxivity measurements also confirmed the presence of the aggregates since the *T*_2_ value of the 1:1 mixture of NPs dropped around 60 % after 30 mins incubation with MMP9 (Table S1).

The ability of the NPs to target the CXCR4 receptor was characterized in vitro by MRI. A CXCR4- and MMP9-expressing cell line (U87.CD4.CXCR4)^3^ was incubated in the presence of the NPs for different periods of time (15, 30 and 60 mins) in order to find an optimal incubation time (Figure 4 B). The maximum decrease in *T*_2_ time was achieved after 30 mins of incubation with the NPs. When CXCR4-positive cells were incubated with targeted NPs, a large decrease in *T*_2_ was observed (Δ*R*_2_/*R*_2only cells_≈160 %; Figure 4 C). However, when either control nontargeted NPs or CXCR4-negative cells (U87.CD4) were used, the *T*_2_ remained almost unchanged (Δ*R*_2_/*R*_2only cells_≈16 %). If an inhibitor of CXCR4 (AMD3100)^20^ was added into the incubation media, the signal recovered to intermediate levels (Δ*R*_2_/*R*_2only cells_≈60 %), thus demonstrating the targeting potential of the NPs. To test whether the cells produced enough MMP enzymes to observe an effect on the *T*_2_ signal, exogenous MMP9 was added into the culture media. The results were very similar to those obtained without extra enzyme, thus indicating that the cells secreted enough MMP2/9 for the reaction to take place (Figure 4 C). Finally, when control NPs without assembling properties, for example, **13**, were incubated with U87.CD4.CXCR4 cells, the contrast of the images (Δ*R*_2_/*R*_2only cells_≈65 %) fell somewhere between the that of the complete targeted NPs and that of the control nontargeted ones.

Before proceeding to in vivo tests, the level of CXCR4 expression in U87.CD4.CXCR4 tumor xenografts in vivo was analyzed by Western blot assays. The results confirmed high levels of CXCR4 expression in these tumors (Figure S10 A). To gauge the maximal tumor signal enhancing capability of the NPs, they were preassembled in an Eppendorf tube in the presence of MMP9. This solution was then injected intratumorally into U87.CD4.CXCR4 xenografts implanted in BALB/c nude mice (Figure S10 B). The mice were imaged before and after injection, and *T*_2_-weighted images acquired from the tumor showed a localized black spot at the injection site with a global decrease in signal intensity in the tumor of around 25 %.

Next, mice bearing tumors were intravenously injected with a mixture of the two populations of NPs in the same needle. Results from mice (*n*=3) injected with targeted IONPs revealed that the *T*_2_ relaxation time of the entire tumor had decreased by approximately 14 % at 4 h after injection (Figure 5 A,C,D). Control nontargeted NPs and saline injections did not produce any significant change in tumor contrast after 4 h (Δ*T*_2_∼0.4 %). Signal measurements from the body *T*_2_-w images showed a decrease in the ratio of liver-to-brain signal both with targeted and control nontargeted NPs, thus confirming the accumulation of NPs in the liver area (Figure S10 C,D). To corroborate the MRI findings, the animals were euthanized after imaging and their organs harvested and analyzed for Fe content (Figure 5 B). The amount of Fe in the tumors of animals injected with the complete NPs was nearly double that found with nontargeted NPs or the nontreated controls. More interestingly, the Fe concentration values for the individual animals correlated well with the decrease in signal from the *T*_2_ maps (Figure S11 A). An increased amount of Fe in organs such as the liver and spleen was also detected, an effect that is typically observed following the administration of IONP contrast agents.^21^ This increase was more evident when nontargeted NPs were used, a result attributed to the fact that targeted NPs were more efficiently retained within the tumors (Figure S11 B).

**Figure 5:**
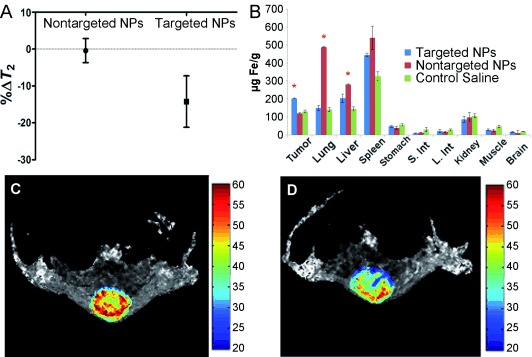
A) Results from the analyses of *T*_2_ maps from a series of spin echo images acquired with different *TE*s. Only the region of interest comprising the tumor was considered for the analyses. B) Iron concentrations in the different organs measured 48 h after the injection of targeted NPs. The results are expressed as the mean of three independent experiments ±the standard deviation. *=statistically different with *p*<0.05, S. Int=small intestine, L. Int=large intestine. Representative tumor *T*_2_ maps are shown from a series of spin echo images acquired with different *TE*s before (C) and 4 h after (D) the intravenous injection of targeted NPs. Color bar scale in milliseconds.

Harvested mouse organs were also used for histological studies after imaging. Staining of the tissues revealed no abnormal pathology of the organs when NPs were injected (Figure S12). After proving aggregation in vitro (Figure 4), a number of in vivo control studies were carried out to ascertain the selectivity of the targeting moiety and of the self-assembly mechanism of Fe accumulation within the tumors. The results (Figure S13) show a significant difference in Fe concentration in tumor between nontreated animals and any of the treated animals (at least a 50 % decrease in Fe concentration in the treated animals; “treated”=inhibitor injection for three days prior to NP contrast agent injection, “non-treated”=NP contrast agent injection only). Both the inhibition of CXCR4 and the inhibition of MMP enzymes decreased the Fe concentrations in the tumors, after the injection of the IONPs, to levels equal to or lower than those of animals in which no contrast agent was injected. Histology of these tumoral tissues further supports these findings (Figure S14). Prussian blue staining for Fe deposits showed the presence of localized increased Fe concentration in tumors of nontreated mice (with sizes up to ca. 10 μm), while in the treated animals, Fe was scarce. These results show that, as proposed, the decrease in MRI signal detected with our probes comes from the combination of a targeted strategy and the response of the probes to MMP enzymes.

Given that our NP system detects both MMP and CXCR4 expression, we have been careful to design the system to reduce pharmacokinetic differences between different sets of particles. We acknowledge that systemic co-administration of a set of NPs could introduce co-delivery issues and that, for our approach to be successful, both of the NPs have to distribute to the target equally well. Notably, some of the parameters that are commonly accepted to have a large influence on biodistribution and circulation, for example, size, shape, and surface charge, are very similar for both families of NPs. It can thus be predicted that the particles will show similar behavior when injected in vivo. Nevertheless, potentially different abilities to reach the tumor cannot be completely excluded.

In conclusion, novel IONPs bearing complementary azide and alkyne click moieties were nanoengineered to undergo copper-free [3+2] cycloaddition following MMP cleavage. This effect was supported by *T*_2_ signal enhancement through cluster formation. This work demonstrates the potential of CXCR4 targeting together with MMP triggers and cycloaddition chemistry for enabling the production of efficient and more sensitive cancer MRI in vivo. The work presented shows that more complex NP designs can be used to confer ‘added value’ on probes. In this case, we have focused on a current limitation of MRI technology, namely sensitivity, but the design can also be extrapolated to other applications, such as drug/gene delivery and targeted/triggered disease treatment. We have demonstrated that more complex design and synthesis can be carried out on NPs with relative ease to enhance their effects both in vitro and in vivo and to elicit an in situ response. This work should pave the way towards the development of further “smart” targeted nanoparticles and, by extension, nanomedicines for a variety of diseases.

## Notes

Supporting information for this article (including experimental details) is available on the WWW under http://dx.doi.org/10.1002/anie.201405442.
