# Supplementary material for: CXCR4-Targeted and MMP-Responsive Iron Oxide Nanoparticles for Enhanced Magnetic Resonance Imaging
Source: Angew Chem Int Ed Engl. 2014 Jul 15;53(36):9550–4. doi: 10.1002/anie.201405442 (PMC4321346; doi:10.1002/anie.201405442)
Supplement: Supplementary file 1 [file anie0053-9550-sd1.pdf]

Angewandte Chemie
Eine Zeitschrift der Gesellschaft Deutscher Chemiker

Supporting Information

© Wiley-VCH 2014

69451 Weinheim, Germany

CXCR4-Targeted and MMP-Responsive Iron Oxide Nanoparticles for Enhanced Magnetic Resonance Imaging**

Juan Gallo, Nazila Kamaly, Ioannis Lavdas, Elizabeth Stevens, Quang-De Nguyen, Marzena Wylezinska-Arridge, Eric O. Aboagye, and Nicholas J. Long**

anie_201405442_sm_miscellaneous_information.pdf

Supporting Information

Experimental Section

General

Preparation of azide nanoparticles

Preparation of alkyne nanoparticles

Nanoparticle characterization

In vitro characterisation

Ex vivo/in vivo characterisation

Supporting figures, tables and data:

Figure S1: Nanoparticle material characterisation

Figure S2: Magnetic measurements from magnetite nanoparticles

Data S3: Nanoparticle material characterization results

Data S4: Nanoparticle functionalization methodology and characterization results

Figure S5: FT-IR characterisation of the azide nanoparticle family

Figure S6: FT-IR characterisation of the alkyne nanoparticle family

Figure S7: ^{1}H NMR spectra of alkyne nanoparticles

Figure S8: MALDI-ToF spectra of the final azide nanoparticles

Figure S9: TEM micrographs of the nanoparticles at different stages

Figure S10: *Ex vivo* characterisation of the tumours, and whole body T_2-weighted MR images before and after the intratumoral injection of the particles

Figure S11: Correlation between Fe content *vs* MR contrast in tumor, and between Fe content in tumor *vs* liver

Figure S12: H&E and Prussian blue stainings of different organs

Figure S13: Fe analysis of treated and non-treated mice

Figure S14: Histological sections of CXCR4 and/or MMP2/9 inhibitor-treated and non-treated tumors stained for Fe with Prussian blue

Table S1: T_1 and T_2 relaxivity rates of different nanoparticle samples

Table S2. Hydrodynamic size and zeta potential of the different NP samples measured in 10 mM PBS at room temperature

Experimental Part

General. All reagents except the cyclopeptide against CXCR4 receptor (Cambridge Peptides), and the non-interfering protein assay (G biosciences) were purchased from Sigma-Aldrich and used as supplied. Fe_3O_4 nanoparticles were prepared according to ref [1]. O-(2-azidoethyl)heptaethylene glycol phosphonooxy-ethyl ester was prepared from O-(2-azidoethyl)heptaethylene glycol according to ref. [2]. Cyclooct-1-yn-3-glycolic acid (COGA) was obtained following ref. [3]. Fourier transform infrared (FT-IR) spectra were recorded using a Perkin Elmer Spectra 100 FT-IR Spectrometer. UV-Vis spectra were recorded using a Perkin Elmer Lambda 25 UV/Vis Spectrometer. A JEOL 2010 transmission electron microscope working at 200 keV was used to image the nanoparticles. Hydrodynamic size studies were performed on a Malvern Zetasizer Nano ZS instrument. MRI images were acquired in a Varian 4.7T instrument.

(I) Preparation of Azide nanoparticles (N_3):

Ligand exchange with O-(2-azidoethyl)heptaethylene glycol phosphonooxy-ethyl ester (1). Before the ligand exchange, the nanoparticle solution (2 mL) was thoroughly washed three times with methanol, and twice with ethanol and acetone, followed by centrifugation for 5 min at 17000 g. Finally the sample was re-suspended in chloroform (4 mL) and mixed with a chloroform solution (4 mL) containing 10 mg (21.08 µmol) of the azide/phosphate molecule. This solution was stirred for 48 h and then the solution was destabilised by the addition of hexane (5 mL). The sample was centrifuged for 5 min at 17000 g, the supernatant discarded and the pellet washed in the same way with hexane. Finally the pellet was re-suspended in chloroform (2 mL) and stored in the fridge until further use.

Partial modification of azide groups into carboxylic groups with cyclooct-1-yn-3-glycolic acid (COGA) (4). 1400 µg (2.2×10^{-6} mmol) of azide functionalised nanoparticles in chloroform were mixed with 42 µg (1.65×10^{-4} mmol) of OCT prepared as described previously .[3] The mixture was shaken at room temperature overnight and the next morning hexane (1.5 mL) was added to destabilise the solution and crush out the particles. The sample was centrifuged at 17000 g for 3 min, the supernatant was discarded and the pellet re-suspended in DCM (250 µL). This process was

repeated twice and finally the nanoparticles were re-suspended in DCM and stored in the freezer until further use.

Coupling of MMP2/MMP9 substrate (6). Before the coupling to the nanoparticles the amine end of the peptide (167 µg, 1.65 x 10^{-4} mmol) was deprotected (commercially supplied as Dnp derivative) by stirring it in a DMF:piperidine 80:20 solution for 90 min. Then, the excess piperidine was removed under vacuum for another 90 min. Meanwhile, in an Eppendorf tube the carboxylic groups on the surface of the nanoparticles were activated using EDC (excess, 10 mg) for 90 mins at room temperature in DCM. After the activation hexane was added (1.5 mL) and the sample was centrifuged at 17000 g for 3 min to remove the excess of EDC. Finally the nanoparticles were re-suspended in DCM (250 µL) and mixed with the DMF solution of the deprotected peptide. This mixture was shaken overnight at room temperature and the next morning the nanoparticles were washed three times with hexane and centrifuged and finally re-suspended in DCM (250 µL). The washings from this reaction were kept to determine the yield of the coupling using a commercial non-interfering protein assay. This step was not carried out for the preparation of control IONPs without self-assembling capabilities.

Coupling of Fmoc-PEG-CO_2H (8). As before, the PEG (139 µg, 1.65 x 10^{-4} mmol) was deprotected under the same conditions, stirred for 90 min in a 80:20 solution of DMF:piperidine. The piperidine was removed under vacuum for 90 min. In the meantime, in an Eppendorf tube the carboxylic groups on the surface of the nanoparticles (-CO_2H terminal group of the MMP substrate peptide) were activated using EDC (excess, 50 mg) for 90 mins at room temperature in DCM. After the activation hexane was added (1.5 mL) and the sample was centrifuged at 17000 g for 3 min to remove the excess of EDC. Finally the nanoparticles were re-suspended in DCM (250 µL) and mixed with the DMF solution of the deprotected PEG. This mixture was shaken overnight at room temperature and the next morning the nanoparticles were washed three times with hexane and centrifuged, and finally re-suspended in DCM (250 µL).

Coupling of cyclic pentapeptide against CXCR4 (10). The sample of nanoparticles from the previous reaction were mixed with an excess of EDC (10 mg) and 9.34 µg (1.00 x 10^{-7} mmol) of the modified cyclopentapeptide. The mixture was shaken overnight at room temperature and the next

morning the nanoparticles were washed three times with hexane, centrifuged and finally re-suspended in DMSO (250 µL). The washings from this reaction were kept to determine the yield of the coupling using a commercial non-interfering protein assay.

(II) Preparation of Alkyne Nanoparticles (≡):

Ligand exchange with 11-aminohexadecanoic acid (2). Before ligand exchange, the original nanoparticle solution (2 mL) was washed extensively three times with methanol, and twice with ethanol and acetone, followed by centrifugation for 5 min at 17000 g. Finally the sample was re-suspended in toluene (4 mL) and mixed with a DMSO solution containing 50 mg (0.24 mmol) of 11-aminohexadecanoic acid (4 mL). This solution was stirred during 48 h and was directly centrifuged for 5 min at 17000 g. The supernatant was discarded and the pellet washed in the same way with a 1:1 mixture of toluene:DMSO. Finally, the pellet was re-suspended in DMSO (2 mL) and stored in the fridge until further use.

Coupling of COGA to the nanoparticle surface (3). 1148 µg (1.79×10^{-6} mmol) of 11-aminoundecanoic acid functionalised nanoparticles were mixed in DMSO with 65.4 µg (3.59×10^{-4} mmol) of OCT prepared as described elsewhere [3] and 10 mg of EDC. The solution was shaken overnight at room temperature and the next morning the nanoparticles were washed three times with acetone and centrifugation. Finally the nanoparticles were re-suspended in DMSO (250 µL).

Partial modification of alkyne groups (5). The nanoparticle solution from the previous reaction was mixed with 32.17 µg (1.34×10^{-4} mmol) of 4-azidophenacyl bromide in DMSO. The reaction was shaken overnight at room temperature. Next morning the nanoparticles were washed with toluene and centrifugation three times and finally re-suspended in DMSO (250 µL).

Coupling of MMP2/MMP9 substrate (7). Before coupling to the nanoparticles the amine functionality of the peptide (144.93 µg, 1.43×10^{-4} mmol) was deprotected (commercially supplied as a Dnp derivative) by stirring it in a DMF:piperidine 80:20 solution for 90 min. Then, the excess of piperidine was removed under vacuum for another 90 min. Meanwhile, in an Eppendorf tube the carboxylic groups on the surface of the nanoparticles were activated using EDC (excess, 50 mg) for

90 mins at room temperature in DMSO. After the activation, toluene was added (1.5 mL) and the sample was centrifuged at 17000 g for 3 min to remove excess EDC. Finally the nanoparticles were re-suspended in DMSO (250 µL) and mixed with the DMF solution of the deprotected peptide. This mixture was shaken overnight at room temperature and the next morning the nanoparticles were washed three times with toluene, centrifuged and finally re-suspended in DMSO (250 µL). The washings from this reaction were kept to determine the yield of the coupling using a commercial non-interfering protein assay. This step was ignored for the preparation of control IONPs without self-assembling capabilities.

Coupling of Fmoc-PEG-CO_2H (9). As before, the PEG (94 µg, 1.43 x 10^{-4} mmol) was deprotected under the same conditions, and stirred for 90 min in a 80:20 solution of DMF:piperidine. The piperidine was removed again under vacuum for 90 min. In the meantime, in an Eppendorf tube the carboxylic groups on the surface of the nanoparticles (-CO_2H terminal group of the MMP substrate peptide) were activated using EDC (excess, 10 mg) for 90 mins at room temperature in DMSO. After the activation, toluene was added (1.5 mL) and the sample was centrifuged at 17000 g for 3 min to remove the excess of EDC. Finally the nanoparticles were re-suspended in DMSO (250 µL) and mixed with the DMF solution of the deprotected PEG. This mixture was shaken overnight at room temperature and the next morning the nanoparticles were washed three times with toluene and centrifugation and finally re-suspended in DMSO (250 µL).

Coupling of cyclic pentapeptide against CXCR4 (11). The nanoparticles from the previous reaction in DMSO were mixed with an excess of EDC (10 mg) and 20 µg (2.14 x 10^{-7} mol) of the modified cyclopentapeptide. The mixture was shaken overnight at room temperature and the next morning the nanoparticles were washed three times with toluene and centrifugation and finally re-suspended in DMSO (250 µL). The washings from this reaction were kept to determine the yield of the coupling using a commercial non-interfering protein assay.

(III) Nanoparticle characterization:

TEM sample preparation. The Fe_3O_4 nanoparticles were deposited in their hexane dispersion (7 µL) onto holey carbon TEM grids with 300 mesh (Agar Scientific, UK) and dried at room temperature.

XRD measurements. X-ray diffraction (XRD) patterns were obtained using Cu-Ka radiation and the samples smeared onto sample holders and scanned.

Magnetic measurements. The saturation magnetization was determined using a vibrating sample magnetometer. The temperature-dependent magnetization of test samples was measured by a superconducting quantum interference device (SQUID). The blocking temperature of test samples was read from the zero-field-cooled and field-cooled curves taken under an applied magnetic field of 100 Oe between 5 and 400 K.

Hydrodynamic size measurements. To measure the evolution of hydrodynamic size of the mixture of nanoparticles 0.01 mM solutions of each final nanoparticle (**11** and **12**) were prepared in 1 mL of MMP9 reaction buffer (50 mM Tris-HCl, pH 7.5, with 10 mM calcium chloride, 150 mM sodium chloride, and 0.05% BRIJ 35). The hydrodynamic size of the particles in this solution was monitored at 25 ^{o}C at 0, 15, 30, 60 and 120 min after the addition of the nanoparticles. The experiment was repeated 3 times. Then, the same concentration of nanoparticles was used in 1 mL of MMP9 reaction buffer and 100 ng/mL of pre-activated MMP9 (overnight, 0.1 mM *p*-aminophenylmercuric acetate (APMA) solution in DMSO) were added. As before, the hydrodynamic size of the particles in this solution was monitored at 0, 15, 30, 60 and 120 mins after the addition of the different components. Again the experiment was repeated three times.

(IV) *In vitro* characterisation:

Phantom imaging of cells incubated with different nanoparticles.

U87 glioblastoma cells stably transfected with CD4 (U87.CD4) or both CD4 and CXCR4 (U87.CD4.CXCR4) were obtained through the NIH AIDS Research and Reference Reagent Program, Division of AIDS, NIAID, NIH from Dr Erman Mandaci. This experimental model is commonly used for the evaluation of drug/probe targeting CXCR4. For the MRI experiments, 5 x 10^5 cells (either Jurkat,

U87.CD4 or U87.CD4.CXCR4) were seeded either in 6 or 24 well-plates 24 h before the experiment. Next day, media was removed, cells were washed twice with warm PBS, and fresh media containing the nanoparticles (50 µg Fe/mL) was added. The cells were then incubated at 37 ^{o}C and 5% CO_2 for a period of time between 15 and 60 mins depending on the experiment. After the incubation period, the media was removed and the cells were washed three times with cold PBS and, in the case of adherent cells, detached either using trypsin or using a scraper. Finally the cells were centrifuged 3 mins at 600 g to form a pellet and these pellets were re-suspended in warm 1% agarose in water (30 µL) and placed immediately in the fridge.

(V) *Ex vivo/in vivo* characterisation:

Western blot.

Tumor samples were excised from animals by dissection and snap-frozen in liquid nitrogen. Lysates were prepared by homogenisation of the tumours in RIPA buffer (Thermoscientific) containing protease inhibitors (Pierce) using Precellys® CK14 lysing kit for Precellys®24 lysis equipment (2 × 25 s at 6500 rpm; Berlin Technologies, France). Proteins of interest were detected via specific antibodies to CXCR4 (Abcam), CD4 (Epitomics) and β-actin (Sigma). Image shown is representative of 3 independent experiments.

MRI and image analysis.

Imaging was performed in a 4.7T horizontal bore DirectDrive Varian MRI system (Palo Alto, CA) equipped with 40 G/cm actively-shielded gradients (VnmrJ 3.1). For imaging the mouse body (biodistribution studies), a 45-mm diameter saddle coil was used in transmit/receive mode and a T_2-weighted fast spin echo (FSEMS) sequence with the following parameters: coronal images, *FOV* = 70 × 35 mm, No slices = 14, slice thickness = 1 mm (zero slice gap), image bandwidth = 100 kHz, *TR* = 2000 ms, TE_{ef}.= 40 ms, N_A = 4, image matrix = 256 × 256, T_A = 6:12 min.

For imaging the tumors and generating the T_2 parametric maps, the 45-mm diameter saddle coil was used in transmit mode and a separate, 20-mm diameter stripline resonator was used as a surface coil [4] to accommodate the tumors. Imaging was performed with a multi-echo multi slice spin echo (MEMS) sequence and the following parameters: axial images, *FOV* = 35 × 35 mm, No slices = 12, slice thickness = 1 mm (zero slice gap), image bandwidth = 208 kHz, *TR*=1320 ms, *TE*. = 12

values (8.95, 17.9, 26.85, 35.8, 44.75, 53.7, 62.65, 71.6, 80.55, 89.5, 98.45 and 107.4 ms), N_A = 2, image matrix = 192 × 192, T_A = 12 min.

During imaging, animals were anesthetised with 2% Isofluorane and respiration and body temperature were monitored via SA physiological monitoring systems (SA Instruments, Stony Brook, NY, USA). Body temperature was monitored by a rectal probe and maintained at 35±1 °C.

For the intratumoral injection of nanoparticles, a 1:1 mixture of the nanoparticles (total Fe concentration 0.75 mg Fe/Kg animal) were used. For the intravenous injection experiments, again a 1:1 mixture of the nanoparticles was used (total Fe concentration of 7.5 mg Fe/kg animal).

For the MRI-based biodistribution studies, the mouse body T_2-weighted images were imported to MIPAV software ((Medical Image Processing, Analysis and Visualisation, National Institutes of Health, US) and then regions of interest (ROIs) were drawn on the mouse liver. ROIs of equal size were then drawn on the mouse brain to which the signal from the liver was then normalised.

T_2 maps were generated in Matlab 10 (The MathWorks®) by fitting the acquired data to the following equation: $S = S_0\exp(-TE/T_2)+N$, where TE is the echo time, T_2 is the transverse relaxation time and N represents the noise floor of the imaging experiments. As in multi echo experiments the first echoes can be of erroneous amplitude due to RF pulse inhomogeneities [5], the first echo (TE = 8.95 ms) was removed from the fitting process. ROIs were then drawn on the T_2 maps to delineate the whole tumor, but with consideration to avoid the edges of the tumor and also contamination from neighbouring tissues and partial volume effects.

Fe concentration studies.

Harvested mice tissues (either after imaging or 4h after IONPs injection) were weighed and digested at 70 ^{o}C for 4 h in concentrated HNO_3 (300 µL). The acid solution (100 µL) was then added into dH_2O (750 µL) and 150 µL of a 1.4M solution of hydroxylamine hydrochloride in 2M HCl. This solution was allowed to react for 30 min and then 100 µL of a 0.01M solution of 3-(2-pyridyl)-5,6-diphenyl-1,2,4 triazine-4',4''-disulfonic acid disodium salt in 0.1M ammonium acetate and 1650 µL of 5M ammonium acetate pH9 were sequentially added. The absorbance of the samples was then measured at 562 nm and the Fe concentration extrapolated from a calibration curve.

Immunohistochemistry.

Tumor, spleen and liver tissues were excised, fixed in formalin, embedded in paraffin, sectioned (5 µm slices) and processed for Prussian blue and hematoxylin and eosin (H&E) staining. Images were captured using an Olympus BX51 microscope.

Small animal experimental models.

All animal experiments were done by licensed investigators in accordance with the UK Home Office Guidance on the Operation of the Animal (Scientific Procedures) Act 1986 (HMSO, London, United Kingdom, 1990) and within guidelines set out by the UK National Cancer Research Institute Committee on Welfare of Animals in Cancer Research [6]. The *in vivo* experimental xenograft models were established by subcutaneous injection of 5 x 10^6 CXCR4-positive human astroglioma cells or their isogenic counterpart (U87.CD4.CXCR4 and U87.CD4, respectively) into 4- to 6-week-old female BALB/c nude mice from Charles River. When xenografts reached approximately 60-80 mm^3 (tumor dimensions were measured using a calliper and tumor volumes were calculated using the ellipsoid formulas, which is best for estimating tumor mass; volume mm^3 = (π/6) x a x b x c, where a, b, and c represent three orthogonal axes of the tumor), mice were administered a single injection of nanoparticles intravenously and MRI scans were performed at the indicated times.

Effect of CXCR4 and/or MMP2/9 inhibition on Fe content *in vivo*.

Mice bearing U87.CD4.CXCR4 xenografts of a size between 60-80 mm^3 were treated daily by intraperitoneal injection for 3 days with 3 mg/kg of the CXCR4 inhibitor, AMD3100 (1,1′-[1,4-Phenylenebis(methylene)]bis-1,4,8,11-tetraazacyclotetradecane octahydrochloride, Sigma; dissolved in 25%DMSO and phosphate buffered saline), 50 mg/kg of a dual MMP2 and MMP 9 inhibitor, MMP2/9 I ((2R)-2-[(4-Biphenylylsulfonyl)amino]-3-phenylpropionic Acid, Calbiochem; dissolved in 25%DMSO and phosphate buffered saline), or a combination of both treatments. One hour after the last dose of drug, targeted IONPs (7.5 mg Fe/kg in phosphate buffered saline) were intravenously injected into the mice, and four hours after the injection, animals were euthanized and their tissues harvested and analyzed as described before.

Supporting figures, tables and data

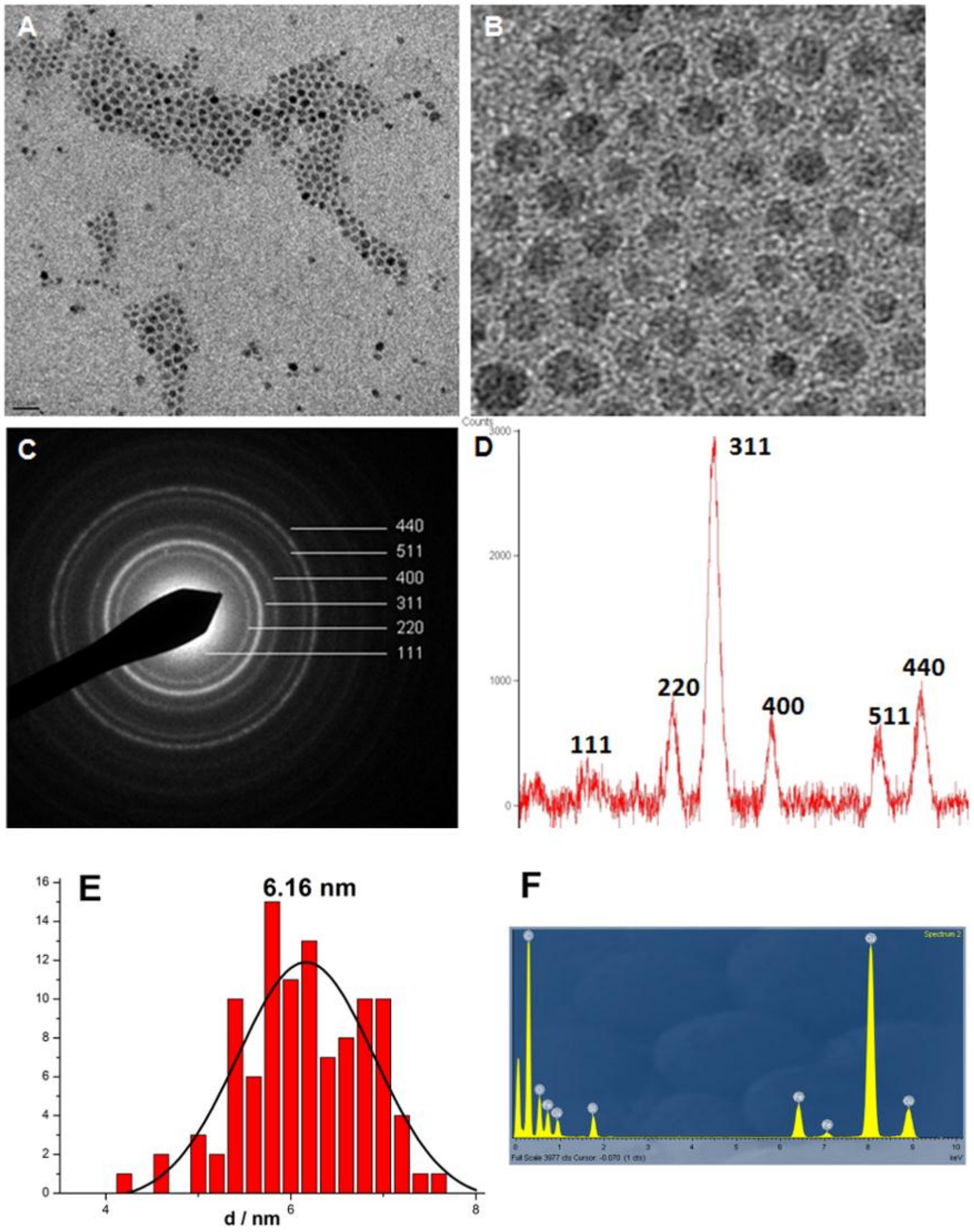

Figure S1. **A**: TEM micrograph of as-prepared Fe_3O_4 nanoparticles (oleic acid/oleyamine capped). (scale bar = 20 nm). **B**: enlarged area from **A.** **C**: SAED showing lattice ring patterns, **D**: XRD diffractogram with indexed peak positions and relative intensities**.** **E**: Size distribution diagram of as prepared Fe_3O_4 nanoparticles. **F**: EDXS spectra of a sample of Fe_3O_4 nanoparticles. Red lines indicate Fe peaks; black lines show Cu and Si peaks coming from the sample holder.

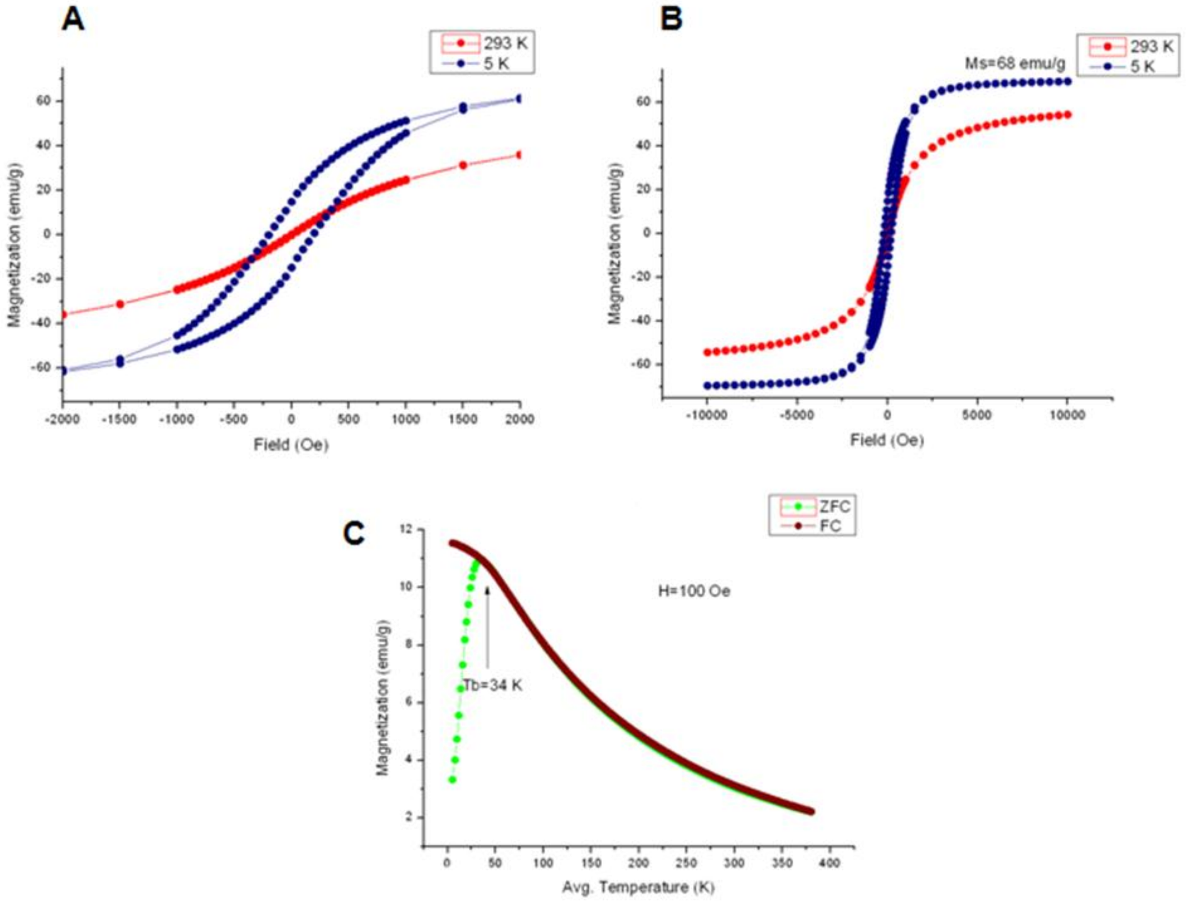

Figure S2. Magnetic measurements of Fe_3O_4 nanoparticles. **A** and **B:** room temperature (red) and low temperature (5 K) (blue) magnetization hysteresis. **C**: zero field cooled (ZFC) and field cooled (FC) magnetization measurements of the same sample.

***Data S3*. Nanoparticle material characterization**

TEM micrographs showed a narrow distribution of spherical NPs, with the average size of ~ 6.1 nm (based on 300 measurements, **Figures S1A and B**). Using TEM, a selected area electron diffraction (SAED) pattern was obtained and the composition of magnetite was confirmed based on measured lattice spacings from the rings in the diffraction pattern, which were comparable to known lattice spacings for bulk Fe_3O_4 along with their respective hkl indexes (**Fig. S1C**). XRD obtained on solid Fe_3O_4 NPs shows several peaks corresponding to the interplanar spacings 111, 220, 311, 400, 511 and 400 comparable to standard peaks (JCPDS, 01-075-0449, Inorganic Crystal Structure Database), confirming the correct reflection peaks for the inverse cubic spinel Fe_3O_4 structure (**Fig. S1D**). The magnetic properties of the sample were measured by SQUID. The normalized saturation (Ms) magnetization for the Fe_3O_4 NPs was measured to be 68 emu/g (**Fig. S2A**) which is comparable to similar sized IONPs (30-60 emu/g) [7]. Zerofield cooled (ZFC) and field cooled (FC) magnetization data measured with a field of 100 Oe in the temperature range 0-400 K were obtained (**Fig. S2C**). In the ZFC measurement, with increasing temperature more and more particles re-orientate their magnetization with the external field and the total magnetization increases, reaching the maximum at the blocking temperature (Tb) which was measured to be 34 K, indicating magnetic interactions between the NPs. These results show that the synthesised IONPs can exhibit superparamagnetic behaviour at room temperature with no hysteresis.

***Data S4*. Nanoparticle functionalization characterization:**

Oleic acid capped NPs **(1,** Figure 2**)** are not dispersible in water and require a second step that transfers them to an aqueous phase. Phosphates are known to bind strongly to Fe_3O_4 surfaces [8], and as such, a phosphate-functionalised PEG ligand bearing an azide group (O-(2-azidoethyl)heptaethylene glycol phosphonooxy-ethyl ester, P-PEG_7-N_3) was synthesized. For the alkyne NPs **(2)**, a commercially available bifunctional (amino/carboxy) molecule was used (11-aminoundecanoic acid, AUA). Both carboxylic and phosphate groups have been shown to bind strongly to Fe_3O_4 surfaces [2, 9]. After the ligand exchange, FT-IR spectrum of N_3-NPs **(3)** showed two strong peaks (2100 cm^{-1} and 1100 cm^{-1}) arising from the N_3 and the phosphate group (Figure S5). For the alkyne-NP **(2),** an indication of successful ligand replacement was a change in solubility, whereby the NPs became soluble in polar solvents such as DMSO and DMF. In these NPs the density of ligands on the particles was previously described to be 11 ligands/nm^2, meaning an average of 1200 ligands per IONP [2]. The next step was reaction with a strained alkyne, cyclooct-1-yn-3-glycolic acid (COGA). In the alkyne-NP family **(4)**, this reaction introduced the group for the final copper-free click chemistry reaction. The carboxylic group in the alkyne molecule was activated *in situ* with EDC (N-(3-dimethylaminopropyl)-N′-ethylcarbodiimide) to react with the amine groups on the surface of the nanoparticles. After the reaction, weak peaks coming from the alkyne could be observed in the IR spectra (2257 and 2130 cm^{-1}, Figure S5). For the other nanoparticles **(5)**, the click chemistry reaction with COGA introduced a new functional group to further modify this system. As the azide groups are still needed in the NPs for self-assembly, the stoichiometry of this reaction was closely controlled. The number of ligands/functional groups on the azide bearing NPs was calculated from thermogravimetric analysis [2] to be around 300, and the stoichiometry was controlled to modify only 100 of them, leaving enough azide units on the NP for the final ’click’ reaction. Again, the IR spectra revealed a new peak in the area of the carboxyl frequencies (1725 cm^{-1}) coming from the free carboxylic group in COGA (Figure S6).

The reaction on the alkyne particles **(6)** followed the same objective as that described above for the azide ones. Copper-free click chemistry was carried out overnight with an azide/bromide molecule (4-azidophenacyl bromide, 4-APB) under closely controlled stoichiometric conditions. ^1H NMR confirmed the presence of a peak in the region of the aromatic protons coming from the benzyl ring introduced (Figure S7).

The final three steps in the preparation of the NPs were common to the two families. For the introduction of a short peptide (to form **7** and **8**), substrate for MMP2/9, the N-terminal amino acid of the peptide was reacted with pre-activated and purified nanoparticles. The yield of this reaction, as measured by the concentration of the peptide in the washings after the reaction, was above 95%. After the successful coupling of the peptide, a bifunctional carboxylic/amine PEG molecule was coupled to the C-terminal amino acid of the MMP substrate (to form **9** and **10**). As before, the amine group of the PEG was reacted overnight with pre-activated and purified nanoparticles. The PEG molecule acts as a spacer between the MMP substrate and the targeting peptide, and at the same time helps avoid clearance by the immune system *in vivo* [10]. Finally, a targeting cyclopeptide directed against CXCR4 was introduced for specific binding to CXCR4 (to form **11** and **12**). The amine group on the side chain of a lysine residue was coupled to the nanoparticle through peptidic chemistry. The yield of this reaction was above 98%, yielding targeted nanoparticles with an average of 10 targeting peptides. The successful preparation of the final ligands was assessed via MALDI mass spectrometry (Figure S8).

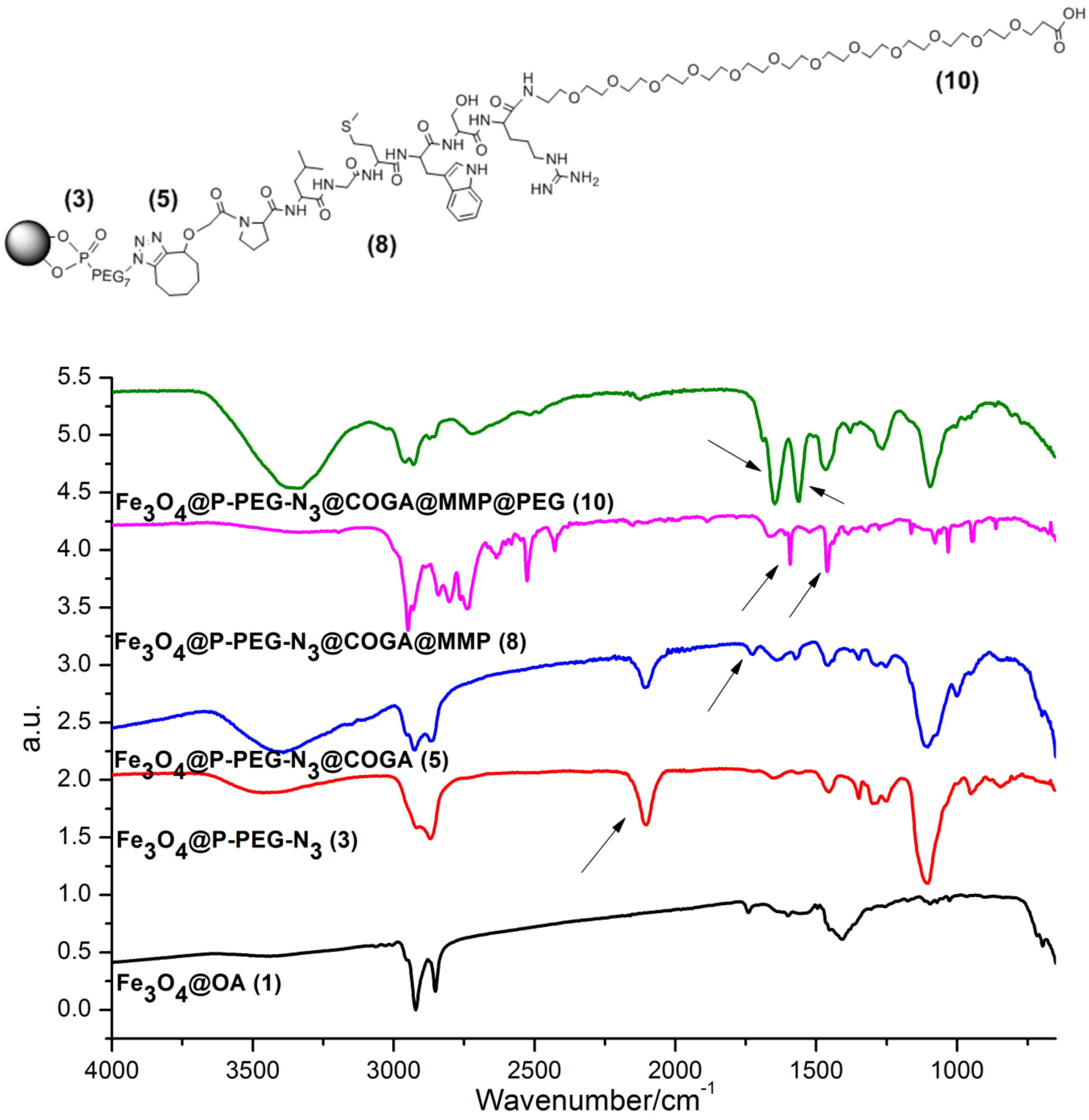

Figure S5. Chemical structure (and respective structural fragments) of the azide-bearing IONPs, and evolution of the FT-IR spectra of the azide-bearing nanoparticles along the synthesis of the final IONPs (relate to Figure 2 in the main text). The distinctive features of each spectra are highlighted with an arrow.

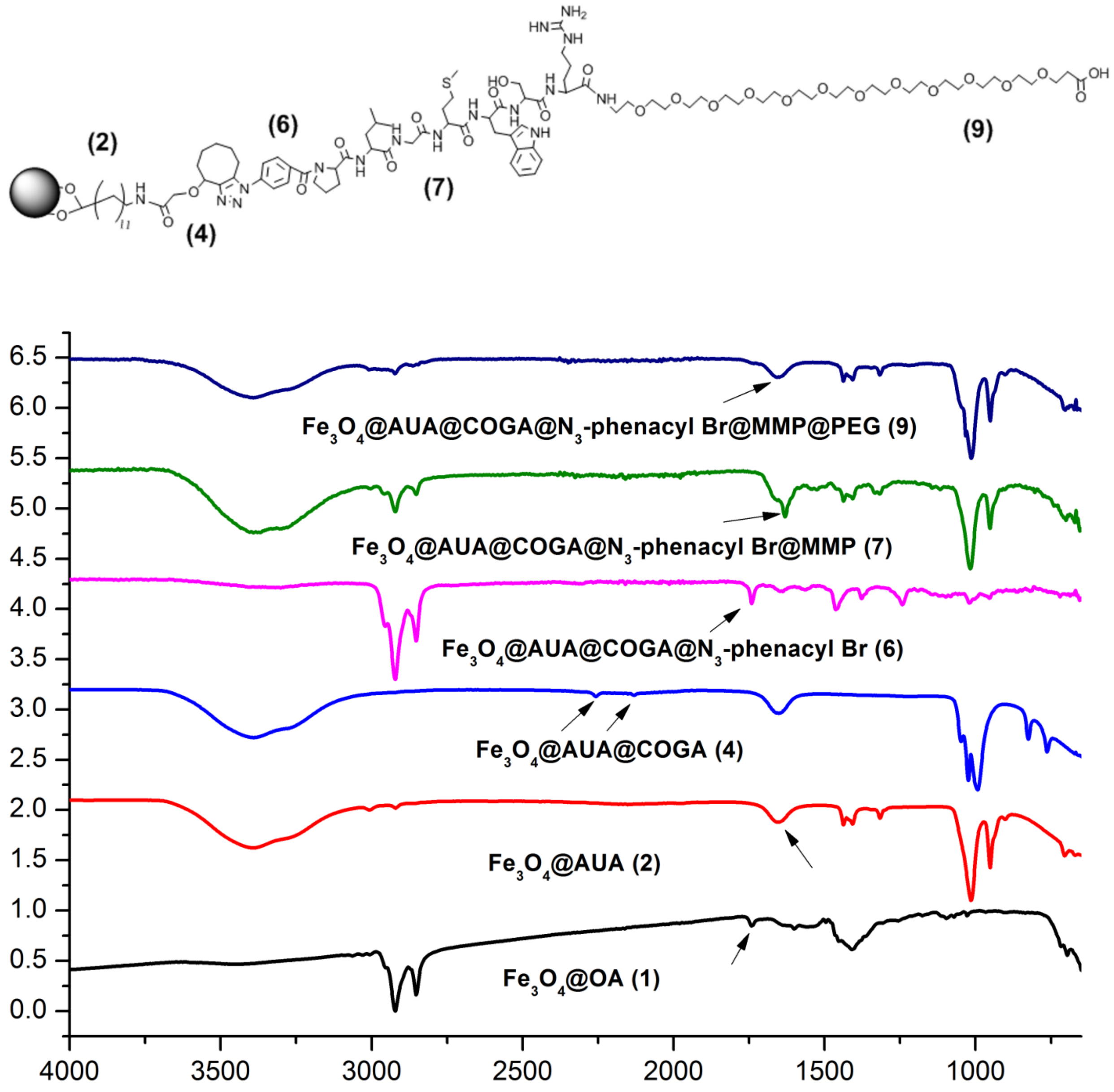

Figure S6. Chemical structure (and respective structural fragments) of the alkyne-bearing IONPs, and evolution of the FT-IR spectra of the alkyne-bearing nanoparticles along the synthesis of the final IONPs (relate to Figure 2 in the main text). The distinctive features of each spectra are highlighted with an arrow.

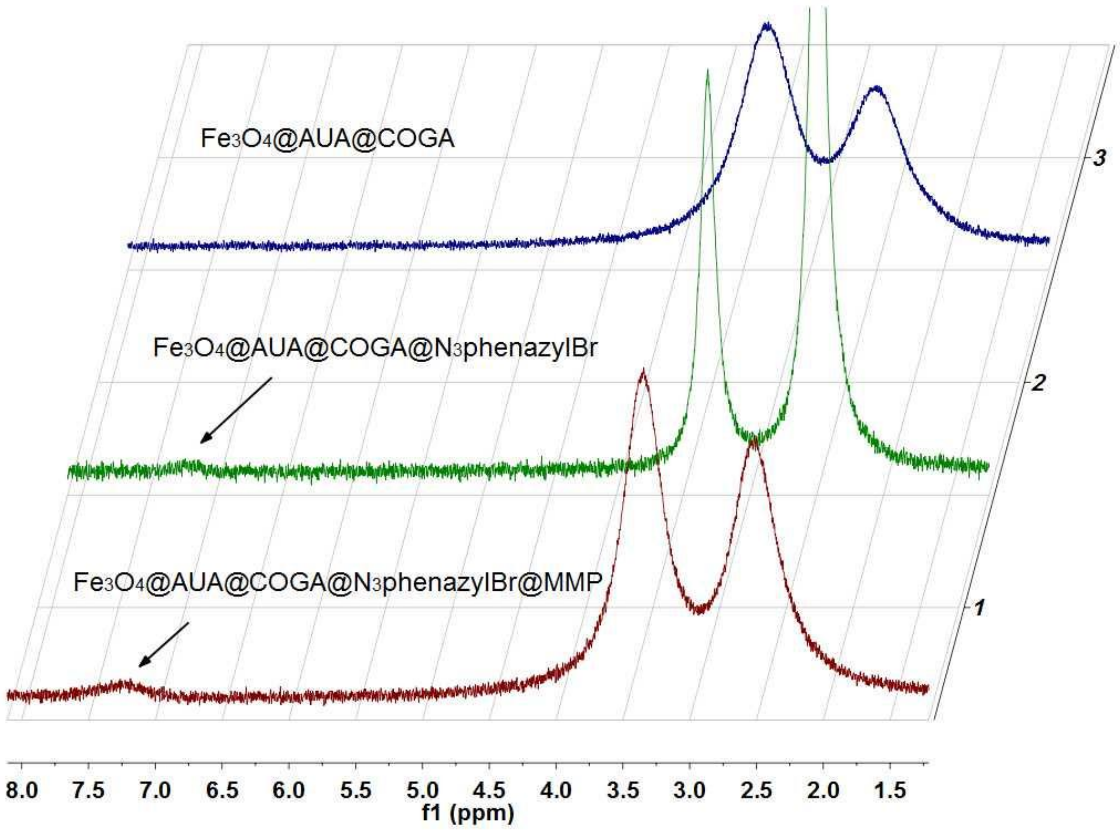

Figure S7. ^{1}H NMR spectra of Fe_3O_4@AUA@COGA (**4**) (blue), Fe_3O_4@AUA@COGA@azidophenazyl bromide (**6**) (green), and Fe_3O_4@AUA@COGA@azidophenazyl bromide@MMP nanoparticles (**7**) (red). Black arrows show the presence of peaks resulting from aromatic protons in the last two samples but not in the first one, confirming the successful coupling of the azidophenazyl molecule. All the spectra were acquired in d_6-DMSO at 500 MHz. The broad bands are a combination of the macromolecular nature of the samples (characteristic of nanoparticulate samples) and the interference of the magnetic field of the nanoparticles and the field of the NMR instrument.

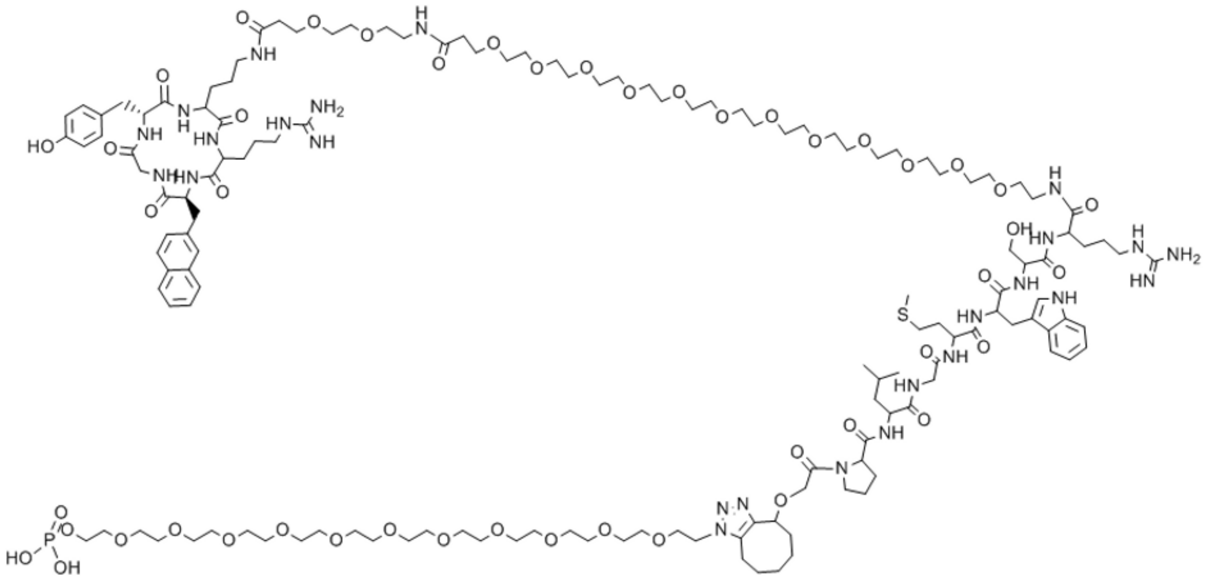

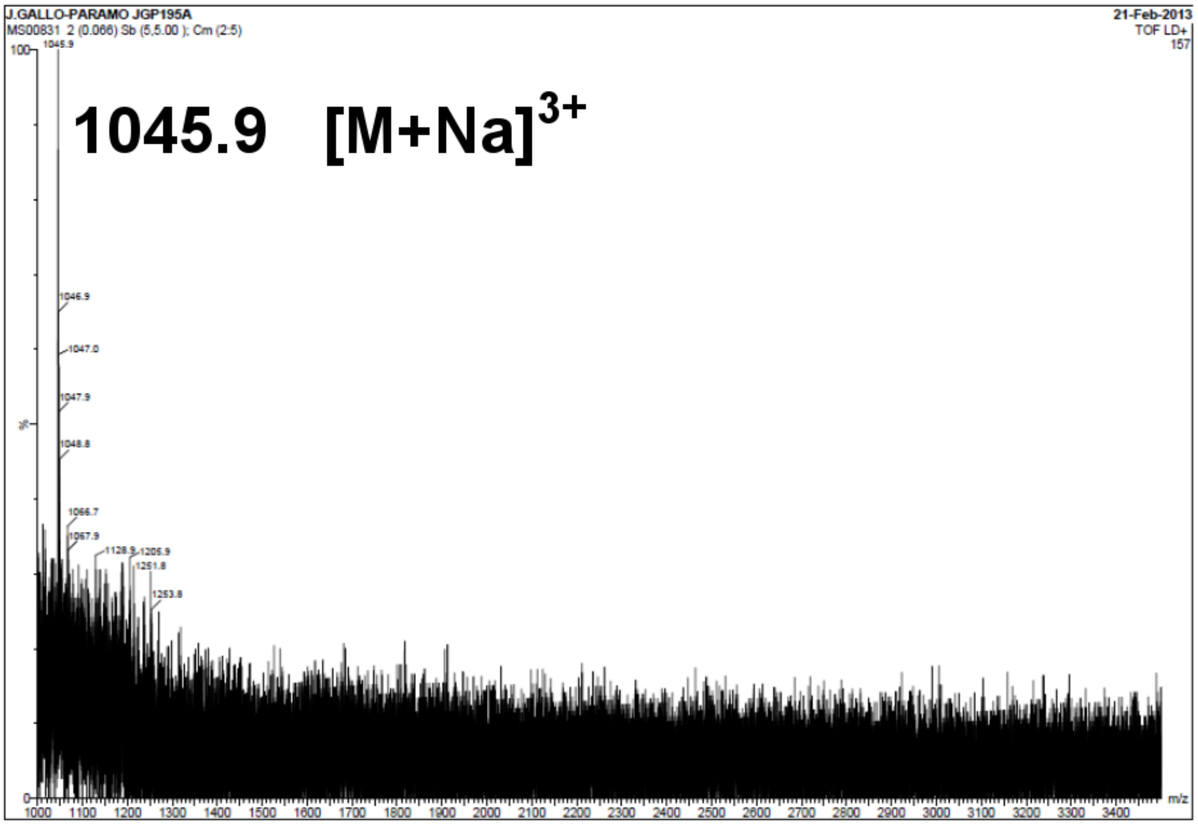

Figure S8. Chemical structure and MALDI-ToF spectra of the final ligand on the azide-bearing IONPs.

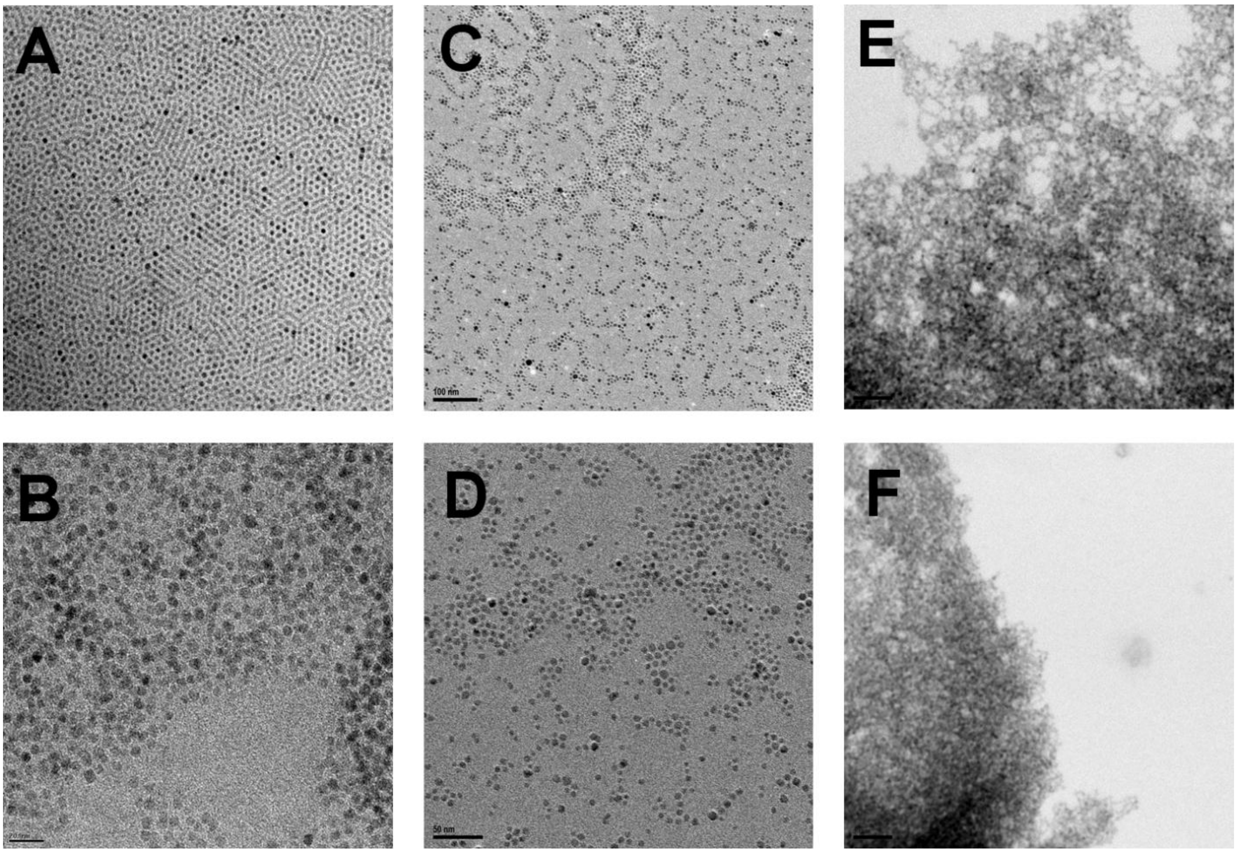

Figure S9. TEM micrographs of the nanoparticles at different stages. **A** and **B**: original oleic acid coated nanoparticles at different magnifications. **C**: Final azide nanoparticles. **D**: Final alkyne nanoparticles. **E** and **F**: overview image of the mixture of azide and alkyne nanoparticles in the presence of activated rhMMP9 enzyme. Scale bars: 100 nm **A**, **C**, **E** and **F**; 20 nm **B**; 50 nm **D.**

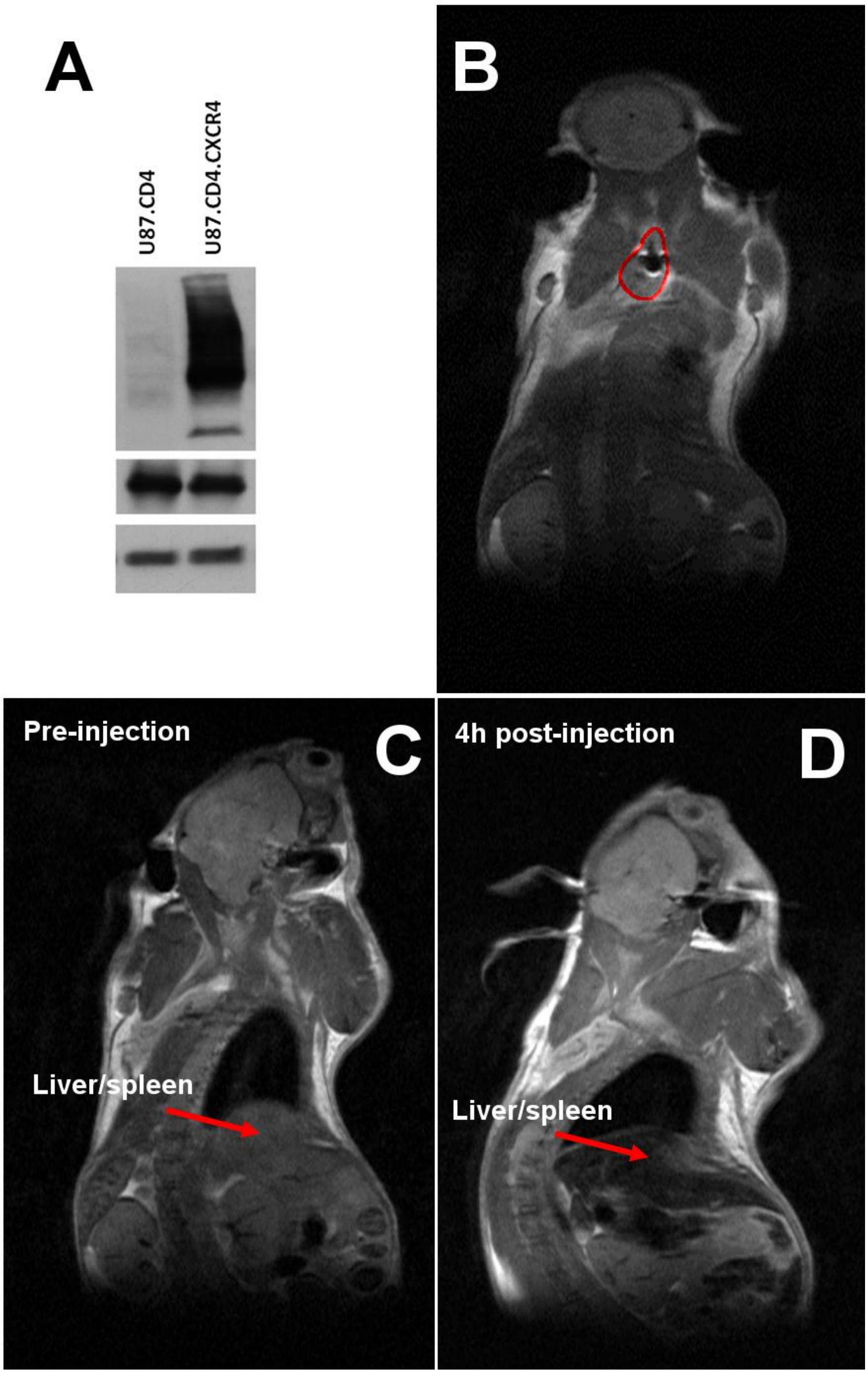

Figure S10. **A**: Protein expression in U87.CD4 tumours as compared to U87.CD4.CXCR4 tumours, as evaluated by western blot (**upper** CXCR4 **middle** CD4 and **lower** β-actin). **B**: whole body T_2-weighted MR image of mouse after injection of 0.75 mg Fe/kg of pre-aggregated targeted nanoparticles **directly** into the tumour (tumour area highlighted in red). **C** and **D**: whole body T_2-weighted images of a mouse before (**C**) and 4 h (**D**) after the injection of 7.5 mg Fe/Kg of targeted nanoparticles. Signal loss in the liver and the spleen due to the accumulation of iron from the nanoparticles is indicated by the red arrows.

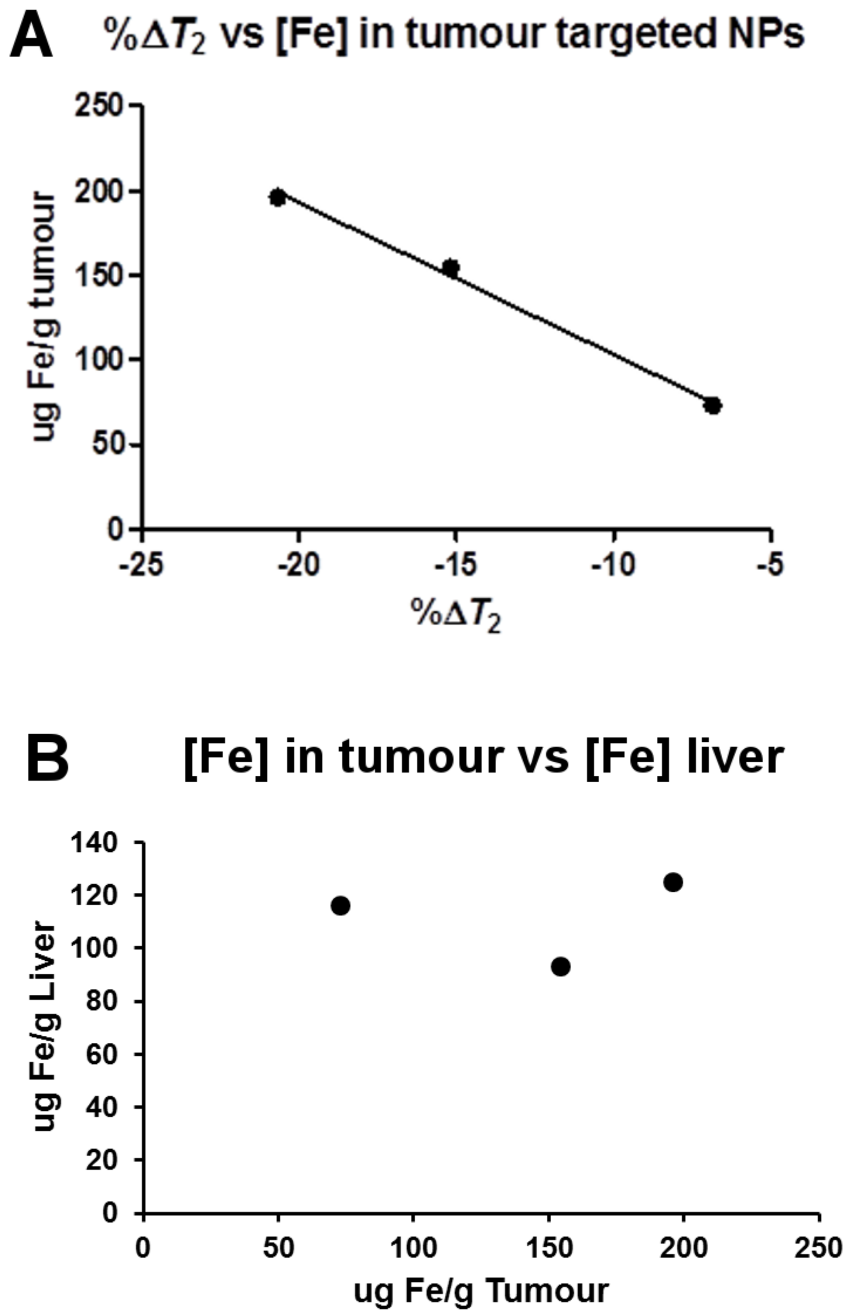

Figure S11. **A**: correlation of the iron concentration in the tumour against the variation of T_2 in the tumour in MR parametric maps. There is good linear correlation (R^2 = 0.98) between both data, supporting the fact that the differences in contrast in the ROI of the tumour are due to the accumulation of the contrast agent. **B**: plot of the iron concentration in the tumour against the iron concentration in the liver. There is no correlation between the data, supporting the fact that the differences in contrast in the ROI of the tumour are not due to differences on the amount of iron injected.

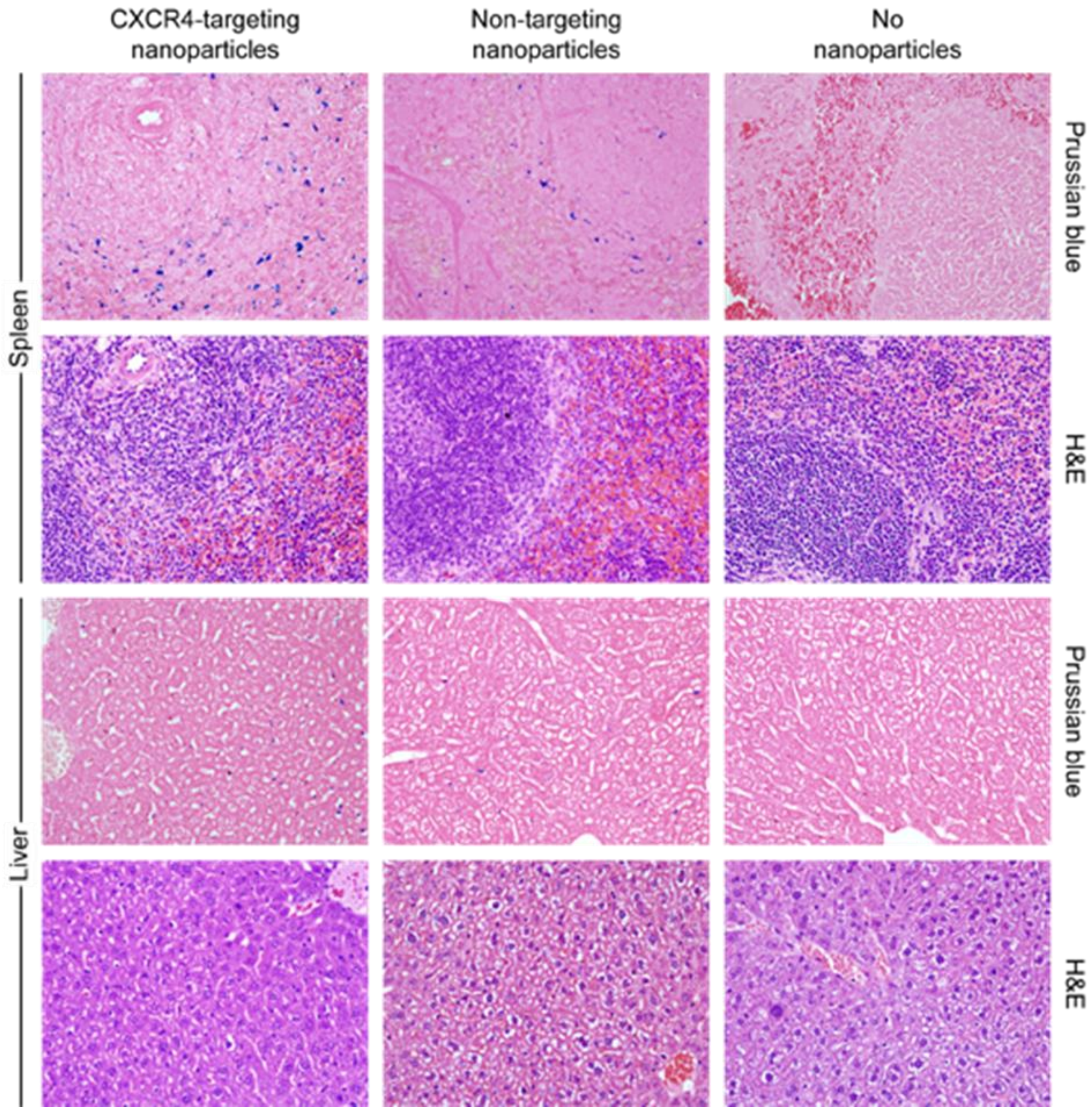

Figure S12. H&E and Prussian blue stainings of tissue from selected organs harvested 48 h after the injection of 7.5 mg Fe/Kg of either targeted or non-targeted contrast agents, and non-injected tissues for comparison.

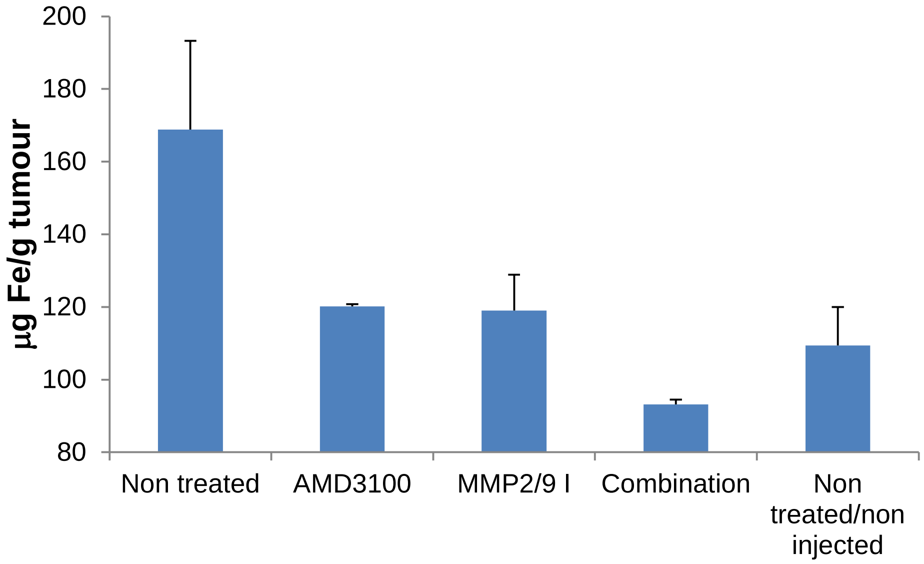

Figure S13. Effect of MMP2/9 and/or CXCR4 inhibition on Fe content in mouse tumors. Mice (n = 3 each) were injected intravenously with 7.5 mg Fe/kg of targeted IONPs (columns 1-4) or received no exogenous Fe (column 5). To assess the effect of MMP or CXCR4 inhibition on exogenous Fe content, mice were untreated (column 1) or pre-treated daily for 3 days with 3 mg/kg of the CXCR4 inhibitor AMD3100 (column 2), 50 mg/kg of dual MMP2 and MMP 9 inhibitor MMP2/9 I (column 3), or both drugs (3 mg/kg of AMD3100 and 50 mg/kg of MMP 2/9 I combined; column 4) prior to injection of the targeted IONPs.

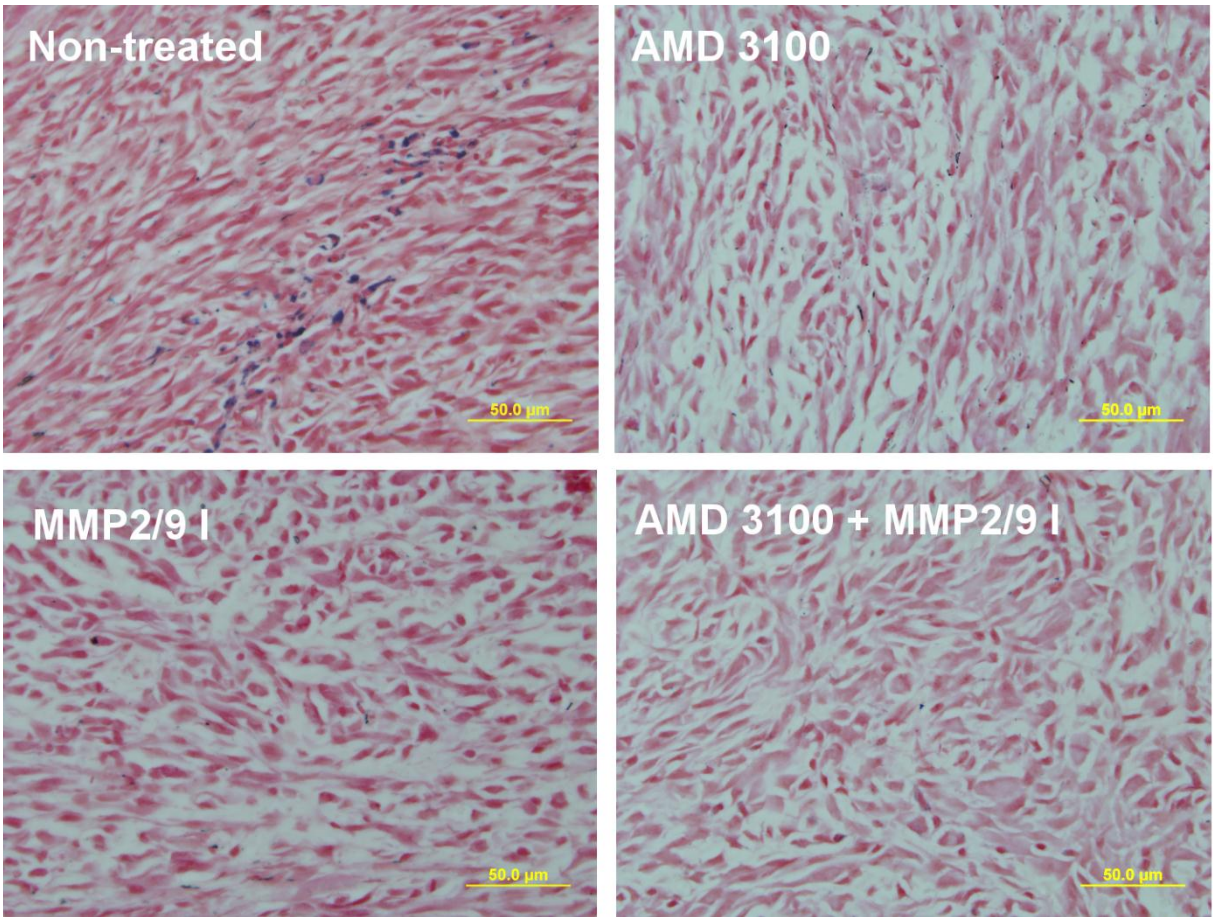

Figure S14. Representative 5 μm thick Prussian blue stained sections of tumoral tissue from mice injected with 7.5 mg Fe/kg of IONPs. Top-left panel, non-treated mouse. Top-right panel, mouse treated once a day for 3 days with the CXCR4 inhibitor AMD 3100. Lower-left panel, mouse treated once a day for 3 days with the dual MMP2 and MMP9 inhibitor MMP 2/9 I. Lower-right panel, mouse treated once a day for 3 days with a combination of both treatments, AMD 3100 and MMP2/9 I. Dark-blue spots indicate Fe accumulation. Magnification x600.

Sample	[Fe] / mM	T_2 / ms	T_1 / ms	R_2 / mM^{-1}s^{-1}	R_1 / mM^{-1}s^{-1}
**N3**	0.2	213 ± 8	2365 ± 62	141.23	1.25
**Alkyne**	0.2	292 ± 11	2456 ± 78	92.31	1.11
**N3 + Alkyne**	0.2	199 ± 7	2125 ± 75	105.65	1.03
**N3 + Alkyne + rhMMP9**	0.2	79 ± 11	922 ± 69	---	---

Table S1. T_1 and T_2 relaxivity rates of the different samples measured in water at room temperature and 400 MHz. Relaxivity (r_1 and r_2) of the different samples measured in water.

Key: N3 = final azide nanoparticle; Alkyne = final alkyne nanoparticle; N3 + alkyne = 1 to 1 mixture of final azide and alkyne nanoparticles; N3 + alkyne + rhMMP9 = 1 to 1 mixture of final azide and alkyne nanoparticles incubated for 30 min in the presence of activated MMP9 enzyme.

Sample	Hydrodynamic size / nm	Zeta potential / mV
**N3**	120 ± 8	-9.4
**Alkyne**	148 ± 10	-5.2

Table S2. Hydrodynamic size and zeta potential of the different NP samples measured in 10 mM PBS at room temperature.

Key: N3 = final azide nanoparticle; Alkyne = final alkyne nanoparticle.

References

[1] S. Sun, H. Zeng, D. B. Robinson, S. Raoux, P. M. Rice, S. X. Wang, G. Li, *J Am Chem Soc* **2004**, *126*, 273-279.

[2] M. A. White, J. A. Johnson, J. T. Koberstein, N. J. Turro, *Journal of the American Chemical Society* **2006**, *128*, 11356-11357.

[3] A. Bernardin, A. Cazet, L. Guyon, P. Delannoy, F. Vinet, D. Bonnaffe, I. Texier, *Bioconjugate Chem* **2010**, *21*, 583-588.

[4] I. Lavdas, H. C. Seton, C. R. Harrington, C. M. Wischik, *Magma* **2011**, *24*, 331-337.

[5] A. P. Crawley, R. M. Henkelman, *Magn Reson Med* **1987**, *4*, 34-47.

[6] P. Workman, E. O. Aboagye, F. Balkwill, A. Balmain, G. Bruder, D. J. Chaplin, J. A. Double, J. Everitt, D. A. H. Farningham, M. J. Glennie, L. R. Kelland, V. Robinson, I. J. Stratford, G. M. Tozer, S. Watson, S. R. Wedge, S. A. Eccles, V. Navaratnam, S. Ryder, N. C. R. Inst, *Brit J Cancer* **2010**, *102*, 1555-1577.

[7] Q. Zhang, C. H. Wang, L. Qiao, H. S. Yan, K. L. Liu, *Journal of Materials Chemistry* **2009**, *19*, 8393-8402.

[8] a) M. A. White, J. A. Johnson, J. T. Koberstein, N. J. Turro, *Journal of the American Chemical Society* **2007**, *129*, 4504-4504; b) U. I. Tromsdorf, O. T. Bruns, S. C. Salmen, U. Beisiegel, H. Weller, *Nano Letters* **2009**, *9*, 4434-4440.

[9] A. Weddemann, I. Ennen, A. Regtmeier, C. Albon, A. Wolff, K. Eckstadt, N. Mill, M. K. Peter, J. Mattay, C. Plattner, N. Sewald, A. Hutten, *Beilstein J Nanotechnol* **2010**, *1*, 75-93.

[10] S. M. Janib, A. S. Moses, J. A. MacKay, *Adv Drug Deliver Rev* **2010**, *62*, 1052-1063.
